# Velocity Curvature Index: a Novel Diagnostic Biomarker for Large Vessel Occlusion

**DOI:** 10.1007/s12975-018-0667-2

**Published:** 2018-10-06

**Authors:** Samuel G. Thorpe, Corey M. Thibeault, Seth J. Wilk, Michael O’Brien, Nicolas Canac, Mina Ranjbaran, Christian Devlin, Thomas Devlin, Robert B. Hamilton

**Affiliations:** 1 Neural Analytics, Inc., 2440 S. Sepulveda Blvd. Suite 115, Los Angeles, CA 90064 USA; 2 Cardiac Biomechanics Group, University of Virginia, Charlottesville, VA USA; 3 Department of Neurology, Erlanger Medical Center, Chattanooga, TN USA

**Keywords:** Ischemic stroke, Transcranial Doppler, Ultrasound, Diagnostic imaging, Large vessel occlusion

## Abstract

Despite being a conveniently portable technology for stroke assessment, Transcranial Doppler ultrasound (TCD) remains widely underutilized due to complex training requirements necessary to reliably obtain and interpret cerebral blood flow velocity (CBFV) waveforms. The validation of objective TCD metrics for large vessel occlusion (LVO) represents a first critical step toward enabling use by less formally trained personnel. In this work, we assess the diagnostic utility, relative to current standard CT angiography (CTA), of a novel TCD-derived biomarker for detecting LVO. Patients admitted to the hospital with stroke symptoms underwent TCD screening and were grouped into LVO and control groups based on the presence of CTA confirmed occlusion. Velocity curvature index (VCI) was computed from CBFV waveforms recorded at multiple depths from the middle cerebral arteries (MCA) of both cerebral hemispheres. VCI was assessed for 66 patients, 33 of which had occlusions of the MCA or internal carotid artery. Our results show that VCI was more informative when measured from the cerebral hemisphere ipsilateral to the site of occlusion relative to contralateral. Moreover, given any pair of bilateral recordings, VCI separated LVO patients from controls with average area under receiver operating characteristic curve of 92%, which improved to greater than 94% when pairs were selected by maximal velocity. We conclude that VCI is an analytically valid candidate biomarker for LVO diagnosis, possessing comparable accuracy, and several important advantages, relative to current TCD diagnostic methodologies.

## Introduction

Acute ischemic stroke (AIS) is the leading cause of long-term disability in the USA, accounting for 87% of ~ 795,000 annual US stroke cases [1]. Intravenous tissue plasminogen activator (IV-tPA) often fails to prevent negative outcomes, even when expediently administered [2, 3]. Recent advances in neurointerventional therapies provide superior treatment options for large vessel occlusions (LVO), but can be limited in effectiveness if not performed within a short window after symptom onset; yielding diminishing therapeutic returns with each subsequent hour [4–6]. Although significant efforts have been undertaken to educate the public and medical responders on stroke symptomology [7], the need for objective, field-deployable diagnostic tools is still great, as rapid identification is critical to ensuring efficient triage and transfer to capable interventional facilities [8]. Unfortunately, prehospital stroke assessment scales currently in use by first responders have been shown to lack sufficient accuracy and reliability [9, 10], causing delays in treatment and diminished access to appropriate care.

Transcranial Doppler (TCD) is a non-invasive ultrasound methodology capable of real-time cerebral blood flow assessment that is also portable and inexpensive. Established clinical indications for TCD are numerous, including cerebral ischemia, sickle cell disease, and vasospasm associated with subarachnoid hemorrhage [11]. TCD examinations to detect stenosed and/or occluded intracranial vessels are routinely conducted as standard of care at many comprehensive stroke centers [12]. Numerous studies comparing TCD examination with radiologic imaging such as computed tomography angiography (CTA) have shown that, when properly performed/evaluated by trained personnel, TCD is a valid and reliable diagnostic tool for detecting LVO [13–17]. However, the specialized training required to inspect flow velocity and interpret waveform morphology across multiple vessels has contributed to TCD being critically underutilized for stroke assessment.

A number of TCD exam methodologies with different criterion for LVO evaluation have been published [13–15]. Typically, cerebral blood flow velocity (CBFV) and power M-mode [18] (PMD) waveforms are obtained for flow through the middle, anterior, and posterior cerebral arteries (MCA, ACA, and PCA) in each cerebral hemisphere, as well as the internal carotid arteries (ICA). Heuristic assessments are then made based on numerous features, including relative velocities, collateral flow, and the presence of pathological waveform morphologies [13, 14]. Such assessments explicitly incorporate information provided by waveform morphology, but in a subjective manner which requires expert evaluators to reliably interpret. More explicit metrics based on inter-hemispheric CBFV comparison have also been shown effective [15]. While objective, reliance on velocity disparity discards much of the information inherent in the full CBFV waveform. Moreover, a number of recent studies have observed quantifiable changes in CBFV morphology associated with various medical conditions [19–23] which are not necessarily associated with significant changes in mean velocity [19].

Since morphological assessment of CBFV waveforms currently requires qualitative interpretation by specialists, development and validation of objective TCD metrics is necessary to enable evaluation by health care practitioners with less formal TCD training. In this work, we evaluate a new candidate diagnostic biomaker [24] by which to evaluate CBFV morphology for the purpose of identifying LVO. Velocity curvature index (VCI; or simply “curvature” in the context of cerebral hemodynamics) provides a quantitative metric which can be used to evaluate a single waveform in isolation, or incorporate information from both cerebral hemispheres. Our goal was to validate the metric and assess diagnostic efficacy and uncertainty relative to CTA. We evaluate metric performance in distinguishing LVO patients from a clinical control group collected in-hospital, analyzing differences with respect to cerebral hemisphere (relative to occlusion), as well as occlusion location, toward the aim of establishing diagnostic utility.

## Materials and Methods

### Patients and Data Acquisition

LVO and in-hospital control (IHC) subject groups were enrolled at Erlanger Health System’s Southeast Regional Stroke Center in Chattanooga, TN, between October 2016 and October 2017. Subjects who arrived at the hospital presenting with stroke symptoms received TCD examinations along with standard care, including pharmaceuticals and CT/A/perfusion imaging. All CTA examinations were performed using a GE Lightspeed VCT 64-section multidetector scanner (GE Healthcare, Milwaukee, WI) with a slice thickness of 0.625 mm, and bolus injection of 70–150 mL of Omnipaque 350 (GE Healthcare, Milwaukee, WI) contrast material (4.0 mL/s). CTA images were reformatted in the coronal and sagittal plane, and 10-mm maximum intensity projection reconstructions were rendered and sent to PACS for review. Occlusion location was determined by the radiologist on call and reviewed/confirmed independently by the authors. TCD examinations were performed during available time between patient testing/treatment, often while CTA results were being processed, and in no way impacted patient care. Subjects for whom an acceptable exam was obtained within 4 h of imaging, and to whom no study exclusion criteria applied (Table 1), were eligible for enrollment in either the LVO group (if CTA confirmed occlusion of the proximal extracranial or terminal intracranial ICA segments, or M1/M2 branches of the MCA), or the IHC group (if no such LVO were detected). Ideally, complete TCD examinations would include scans of the left/right MCA across multiple depths. To be considered acceptable, exams had to include at least one bilateral pair of left/right MCA scans at depths between 45 and 60 mm, each containing at least 15 distinguishable beat waveforms. Data collection and analysis protocols were approved by the University of Tennessee College of Medicine Institutional Review Board (ID: 16-097).

**Table 1:** Subject exclusion criteria

Exclusion criteria	
1. Head CT findings consistent with acute primary intracranial hemorrhage (SAH, ICH, etc.) 2. Hemodynamically unstable patients requiring pharmacological support for hypotension 3. Subjects who underwent partial or full craniotomy 4. Additional intracranial pathologies present (tumor, hydrocephalus, etc.) 5. Anticipated insufficient time to acquire a complete set of scan as described by the protocol 6. Significant hemodynamic pharmacological agent (cocaine, amphetamine, etc.) 7. Subjects who are under arrest for a felony	

### TCD Waveform Recording

A trained technician transtemporally insonated the left/right MCA using 2-MHz handheld ultrasound probes in conjunction with either DWL Doppler Box-X (DWL Inc., USA), or Lucid M1 TCD System (Neural Analytics Inc., USA). Waveform recordings were made in 30-s intervals across multiple depths between 45 and 60 mm. The technician marked the start/stop times of each interval using a custom remote, the output of which was temporally aligned with CBFV envelopes (digitally sampled at 125 Hz) using custom software (Python 2.7). The technician minimized subject movement artifact by holding the probe in one hand, and gently bracing the subject’s head with the other. Often, available nursing staff also assisted in helping brace the subject.

### TCD Waveform Processing

Individual beat waveforms from each interval were extracted using a combination automated beat identification algorithm with manual checking/editing. In this procedure, individual beats were first identified automatically using an internally developed beat extraction tool and displayed to the user for manual confirmation/editing. Detected beats which lacked clear pulsatile structure or deviated anomalously from the group average (usually due to probe displacement) were excluded. The remaining beats were aligned and averaged, resulting in a single representative beat waveform for each interval. Because curvature is a nonlinear function sensitive to small inflections, the resultant average beat waveforms were smoothed via convolution with a 90 ms Hanning window.

For each average beat waveform, denoted *x*, local curvature (*k*) was computed at each time point (*t*_*i*_) via the following discretized expression for unsigned graph curvature:

$$ k\left({t}_i\right)=\frac{\mid {\delta}^2\left[x\right]\left({t}_i\right)\mid }{{\left(1+{\left(\varDelta \left[x\right]\left({t}_i\right)\right)}^2\right)}^{\frac{3}{2}}} $$

where Δ and *δ*^2^ are the first order (backward) and second order (central) finite difference equations:

$$ \varDelta \left[x\right]\left({t}_i\right)=x\left({t}_i\right)-x\left({t}_{i-1}\right) $$

$$ {\delta}^2\left[x\right]\left({t}_i\right)=x\left({t}_{i+1}\right)-2x\left({t}_i\right)+x\left({t}_{i-1}\right) $$

**Fig. 1:**
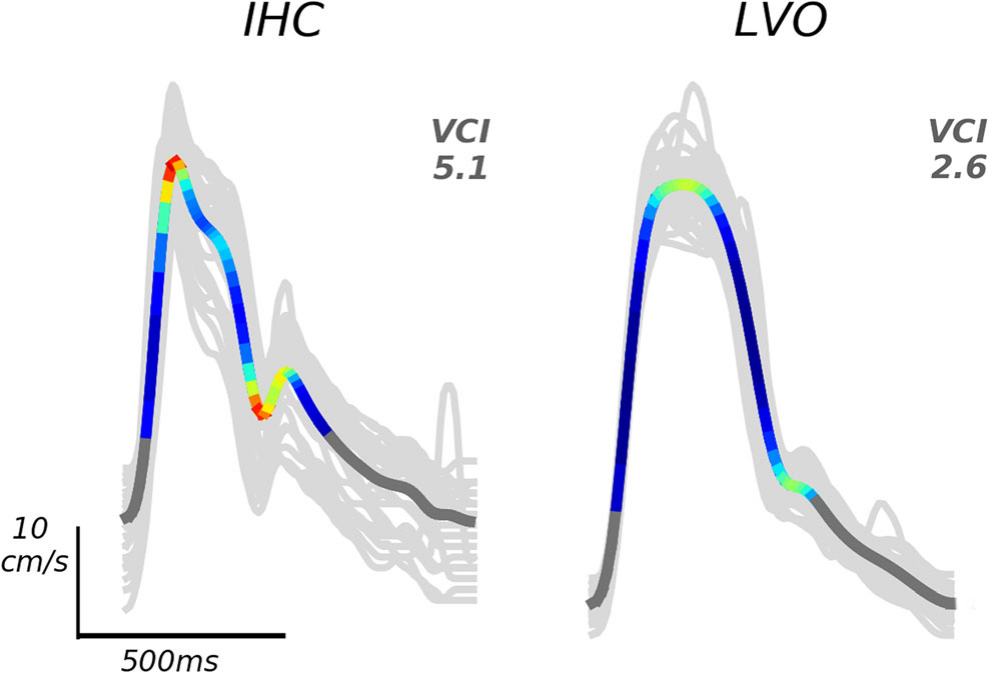
Example average beat waveforms from IHC (left), and LVO (right) groups are depicted with local curvature indicated by color. Areas of high curvature are shown in hot colors (red/yellow), whereas low curvature is indicated by cool colors (blue/green). Dark gray areas indicate time points not included in the beat “canopy,” where velocity is less than one quarter of its total diastolic-systolic range. Light gray traces depict individual beats over which each average was taken

A single VCI metric for each waveform was then obtained by summing local curvature over all time points comprising the beat “canopy,” defined as the set of points wherein velocity exceeds one quarter of its total diastolic-systolic range, i.e., all *t* such that *x*(*t*) ≧ *x*(*t*_*d*_) + 0.25(*x*(*t*_*s*_) − *x*(*t*_*d*_)), where *t*_*d*_ and *t*_*s*_ represent time points corresponding to diastolic minimum and systolic maximum, respectively (Fig. 1). The specification of 0.25 represents a free parameter in the metric computation. High values for this parameter risk excluding important morphological dynamics, whereas low values risk degraded signal due to inclusion of envelope noise typically prevalent near the diastole. Here, we have fixed the parameter based on our empirical estimate of the average onset of diastolic decay. In practice, the results of the following analyses do not depend critically on this value in the range we have specified (see discussion in the “Pair Selection and Free Parameters”section).

### Statistical Analysis of VCI

In the manner described above, we obtained VCI distributions for each subject corresponding to multiple recordings spanning depths between 45 and 60 mm over both cerebral hemispheres. To assess variability across groups, we tested whether group VCI were drawn from distributions with the same underlying mean. Group VCI distributions were obtained by averaging individual subject VCI across depths. Since the resultant group variables were not normally distributed, we bootstrapped empirical *p* values representing the likelihood of observed differences assuming common underlying means. This was accomplished by iteratively resampling each group with replacement [25], each time taking the mean, resulting in an empirical distribution of 10,000 bootstrap samples describing the variability in expected VCI for each group. To assess significance between two groups, these distributions were subtracted pair-wise, resulting in a single distribution of expected group mean differences, from which empirical *p* values were computed as the minimum proportion of difference samples of the same sign (positive or negative). Empirical *p* values below the specified critical value of 0.01 were deemed significant. To test whether VCI depends on cerebral hemisphere relative to occlusion location, we split the LVO group recordings for each subject into ipsilateral and contralateral subgroups and compared VCI by bootstrapping empirical *p* values as previously described.

Since the presence and location of occlusion cannot be known a priori in real-world applications, we tested a paired version of the VCI metric which uses information from both hemispheres. For this analysis, the applicable space for each subject is the set of all possible *pairs* of bilateral recordings. VCI for each pair was taken as the minimum of the two VCI within the pair. This procedure effectively guarantees that if an occlusion is present in either cerebral hemisphere, ipsilateral VCI factors into the assessment. To assess variability across groups, we averaged paired-VCI across pairs for each subject and tested whether the resultant group variables were drawn from distributions with the same mean, using the same previously described procedures. Additionally, to test whether VCI depends on which vessel was occluded, we split the LVO subjects into subgroups corresponding to M1 and ICA occlusions, and compared VCI by bootstrapping empirical *p* values as previously described.

Finally, we aimed to test whether VCI performance might be improved by an informed selection of bilateral pairs. In practice, waveforms with the highest measured velocities for a given vessel are assumed to most accurately reflect reality, owing to the fact that Doppler velocities scale with the cosine of the incident angle between the ultrasound beam and underlying blood flow [26]. In line with this reasoning, we selected pairs for each subject with maximal mean velocity measured across depths for each hemisphere and tested whether the resultant VCI from Max Velocity Pairs (MVP-VCI) were drawn from distributions with the same mean, using the same previously described procedures. An analogous subgroup analysis comparing MVP-VCI for M1 and ICA occlusions was also performed for comparison.

### VCI Receiver Operating Characteristic

To assess the degree of separability between LVO and control group VCI distributions in a manner incorporating uncertainty from all individual subject recordings, we bootstrapped empirical ROC curves and tested the significance of differences between associated area-under-curve (AUC) distributions. This was accomplished by iteratively sampling curvature from a randomly chosen recording for each subject, and each time computing the associated ROC curve between LVO and IHC groups, resulting in a distribution of 1000 ROC curves and associated AUC measures. For each iteration, the ROC curve and associated AUC were computed along with the sensitivity and specificity at the maximal Youden J-Statistic threshold [27]. Analogous methods were used for obtaining ROC curves and associated AUC comparing ipsi/contralateral LVO subgroups and controls, as well as paired-VCI comparisons between subject groups (with the caveat that VCI for each subject was iteratively sampled from a randomly chosen *pair*). Additionally, AUC distributions comparing ipsi/contralateral VCI to controls were tested for significant differences. For each group comparison, empirical distributions were subtracted pair-wise to obtain a single distribution representing expected AUC difference, from which empirical *p* values were again computed as the minimum proportion of difference samples of the same sign.

## Results

### Subject Demographics

A total of 88 subjects with sufficient initial screenings were obtained at Erlanger Medical Center, of which 50 and 38 were initially enrolled in the LVO and IHC groups, respectively. Three LVO subjects were discontinued (subject either expressed desire to discontinue or was transferred or died before enrollment was completed). An additional 14 LVO and 5 IHC subjects were subsequently excluded due to disqualifying criteria unknown at the time of enrollment. In total, the current analyses included 33 LVO and 33 IHC subjects, with 44 (19 IHC) acquired using the DWL system, and 22 (14 IHC) acquired using the Lucid M1 System. Results of all statistical tests presented herein were identical when performed individually on exams from each system. Patient demographic and occlusion location information is provided in Tables 2 and 3.

**Table 2:** Subject demographics. NIHSS denotes National Institute of Health Stroke Scale. For age and NIHSS, group means are reported with standard deviation in parentheses. F/M in reported gender imply female/male, respectively

Subject demographics and medical history						
	Age	Gender	NIHSS	Hypertension	Diabetes	Previous stroke
*LVO*	67 (15.7)	16F, 17M	16.8 (6.6)	21	1	2
*IHC*	56 (16.3)	13F, 20M	1.9 (2.0)	14	0	1

**Table 3:** Occlusion location breakdown for the LVO subject group. peICA and tiICA denote proximal extracranial ICA, and terminal intracranial ICA, respectively. Subjects with multiple occlusions included one with dual occlusion of both the M1 and ICA (same hemisphere), and another with bilateral occlusions of both ICA, in addition to an M2 occlusion

Occlusion location					
Total	M1	M2	peICA	tiICA	Multiple
33	20	3	6	2	2

### Single-Sided VCI

**Fig. 2:**
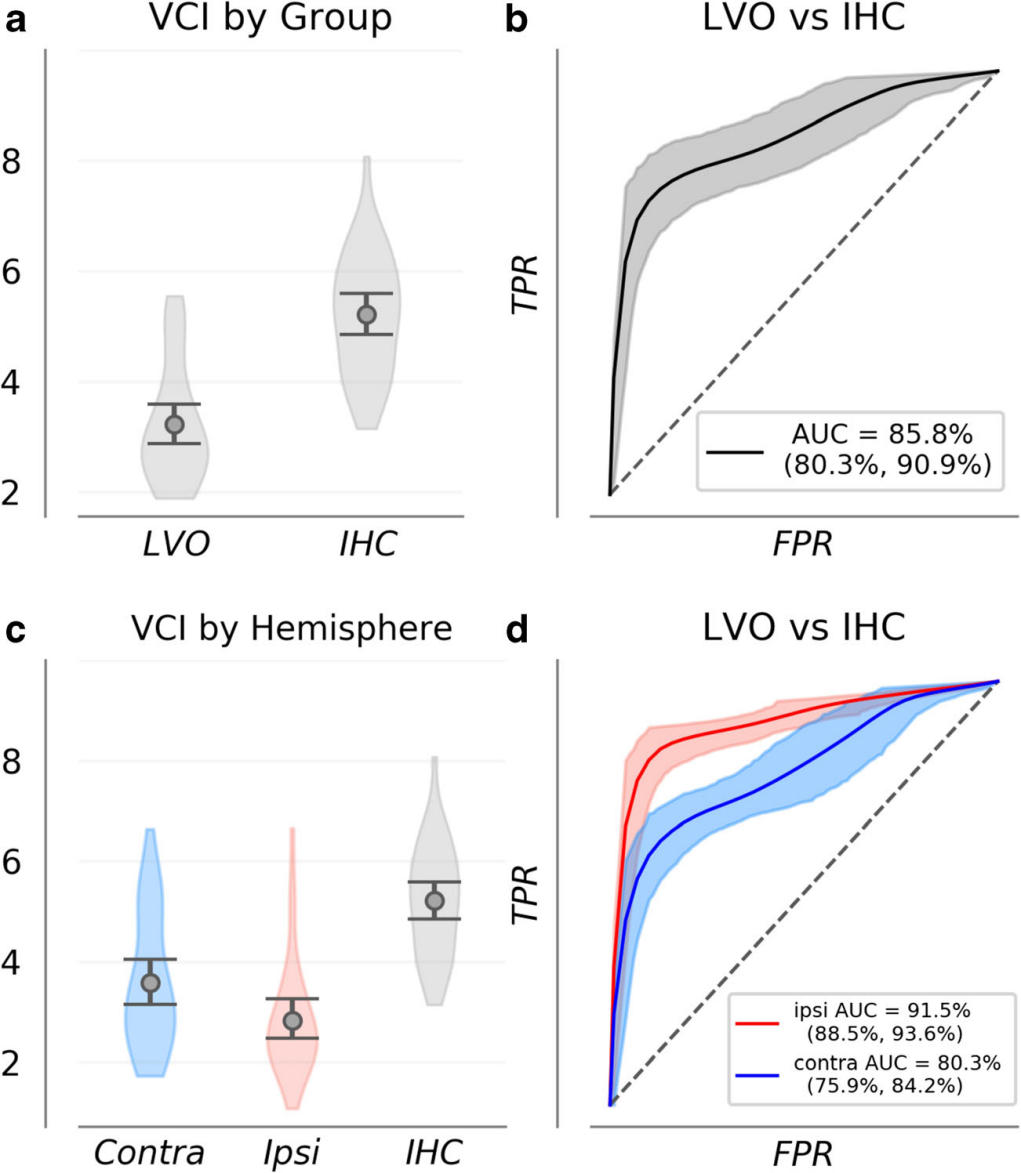
VCI distributions are shown for each group (**a**, **c**) along with bootstrapped ROC curves depicting group separability and empirical uncertainty (**b**, **d**). **a** shows VCI averaged over all recordings for each subject, combined across hemispheres. In **c**, the LVO group is separated by hemisphere relative to occlusion. Together, these curves demonstrate that VCI measurements from both hemispheres provide information concerning the presence of LVO. However, ipsilateral measurements are significantly more information rich. Light gray (**b**), and light blue/red (**d**) regions depict 95% confidence intervals on the true positive rate as a function of false positives

VCI distribution means were 3.2 (CI = 2.9–5.4) for LVO subjects and 5.2 (CI = 4.9–5.6) for IHC (Fig. 2a). Significance testing confirmed VCI to be greater for IHC relative to LVO (*p* ≪ 0.001). Observed means for ROC-AUC distributions (Fig. 2b) were 85.8% (CI = 80.4–91.3%). Associated sensitivity and specificity distributions had means of 72.6% (CI = 60.6–84.8%) and 93.2% (CI = 81.8–100%).

When broken into subgroups according to hemisphere relative to occlusion location, ipsilateral and contralateral subgroups were comprised of data from 32 subjects each (one LVO subject was excluded due to bilateral ICA occlusions). Observed VCI distribution means were 2.8 (CI = 2.5–3.3), and 3.6 (CI = 3.2–4.1) for the ipsilateral and contralateral subgroups, respectively (Fig. 2c). Significance testing confirmed mean VCI to be greater for the contralateral subgroup relative to ipsilateral (*p* = 0.006) and confirmed both LVO subgroups to be significantly lower than IHC (*p* ≪ 0.001 for both tests). For ROC comparisons between LVO subgroups and the IHC group (Fig. 2d), observed AUC means were 91.5% (CI = 88.6–93.8%), and 80.2% (CI = 75.8–84.4%) for ipsilateral and contralateral, respectively, with significance testing confirming ipsilateral VCI as more separable from IHC than contralateral (*p* ≪ 0.001).

### Paired-VCI

**Fig. 3:**
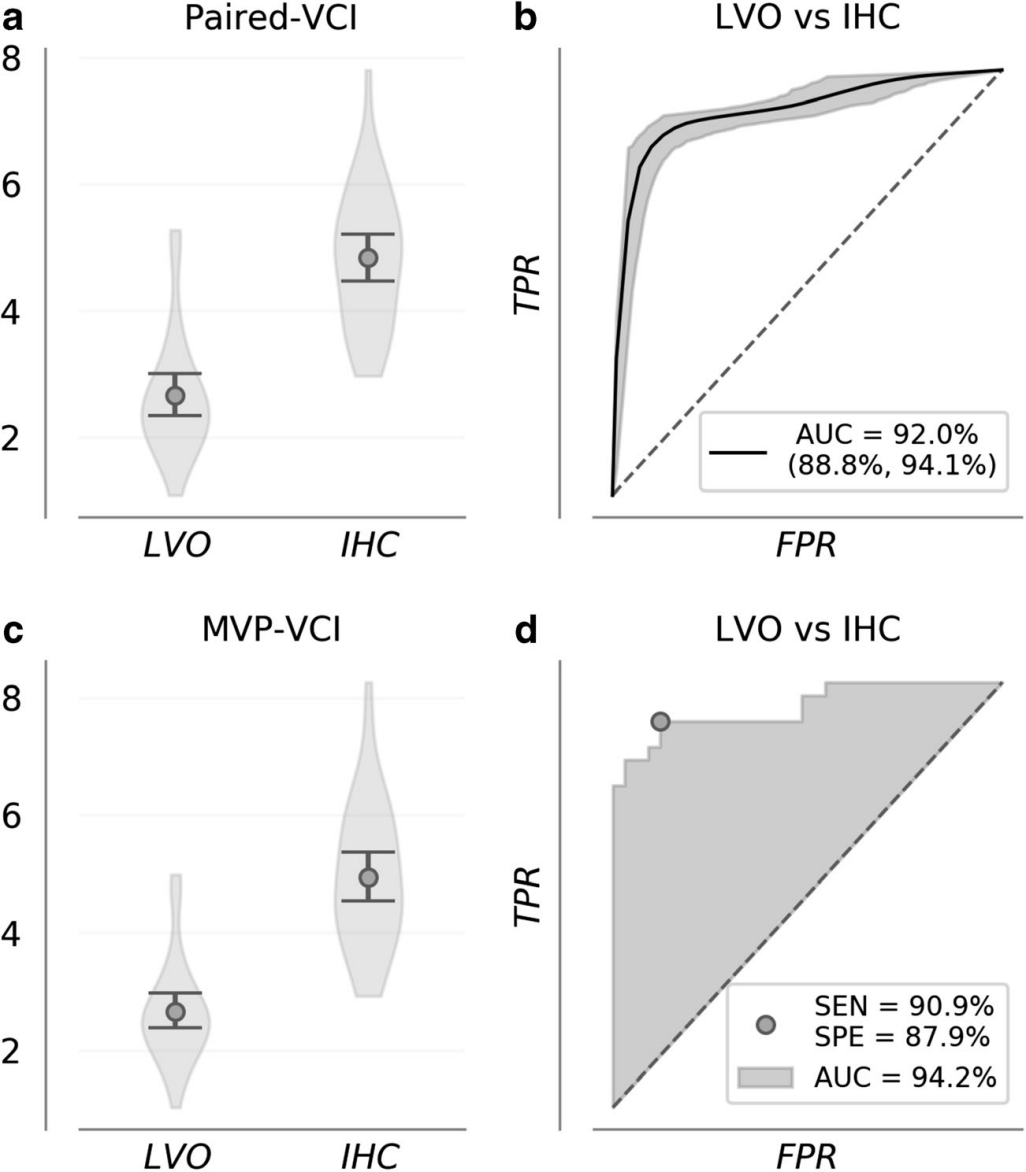
Paired-VCI distributions are shown for each group (**a**) along with bootstrapped ROC curves depicting group separability and uncertainty (**b**). **a** shows paired-VCI averaged over all bilateral recording pairs for each subject. Paired comparison increases signal efficacy by enforcing evaluation of the ipsilateral hemisphere, regardless of whether occlusion location is known. **c**, **d** show analogous distributions and ROC curves wherein pairs for each subject are chosen by maximal velocity across depths for each hemisphere, with sensitivity and specificity indicated at maximal Youden’s J-statistic threshold. In 3D, AUC for the ROC curve comparing MVP-VCI of the LVO to controls outperforms the upper tail of the corresponding 95% confidence intervals shown in **b**, demonstrating improvement of the max velocity criterion relative to random pairs

Paired-VCI distribution means were 2.7 (CI = 2.4–3) for LVO subjects and 4.8 (CI = 4.5–5.2) for IHC (Fig. 3a). Significance testing confirmed paired-VCI to be greater for IHC relative to LVO (*p* ≪ 0.001). Observed means for ROC-AUC distributions (Fig. 3b) were 92% (CI = 89–94.1%), and associated sensitivity and specificity distributions had means of 84.7% (CI = 75.8–90.9%) and 93.3% (CI = 84.8–100%).

When pairs were selected according to maximal velocity, MVP-VCI distribution means were 2.7 (CI = 2.4–3) for LVO subjects and 5 (CI = 4.6–5.4) for IHC (Fig. 3c). Significance testing confirmed MVP-VCI to be greater for IHC relative to LVO (*p* ≪ 0.001). The ROC curve comparing LVO to IHC showed AUC of 94.2%, with sensitivity and specificity of 90.9% and 87.9% at the maximal Youden’s J-Statistic threshold (Fig. 3d). Moreover, AUC associated with MVP-VCI pairs was observed to be greater than the upper tail of the corresponding confidence intervals noted in Fig. 3b, suggesting max velocity pairing represents a significant performance improvement over random bilateral pairs.

**Fig. 4:**
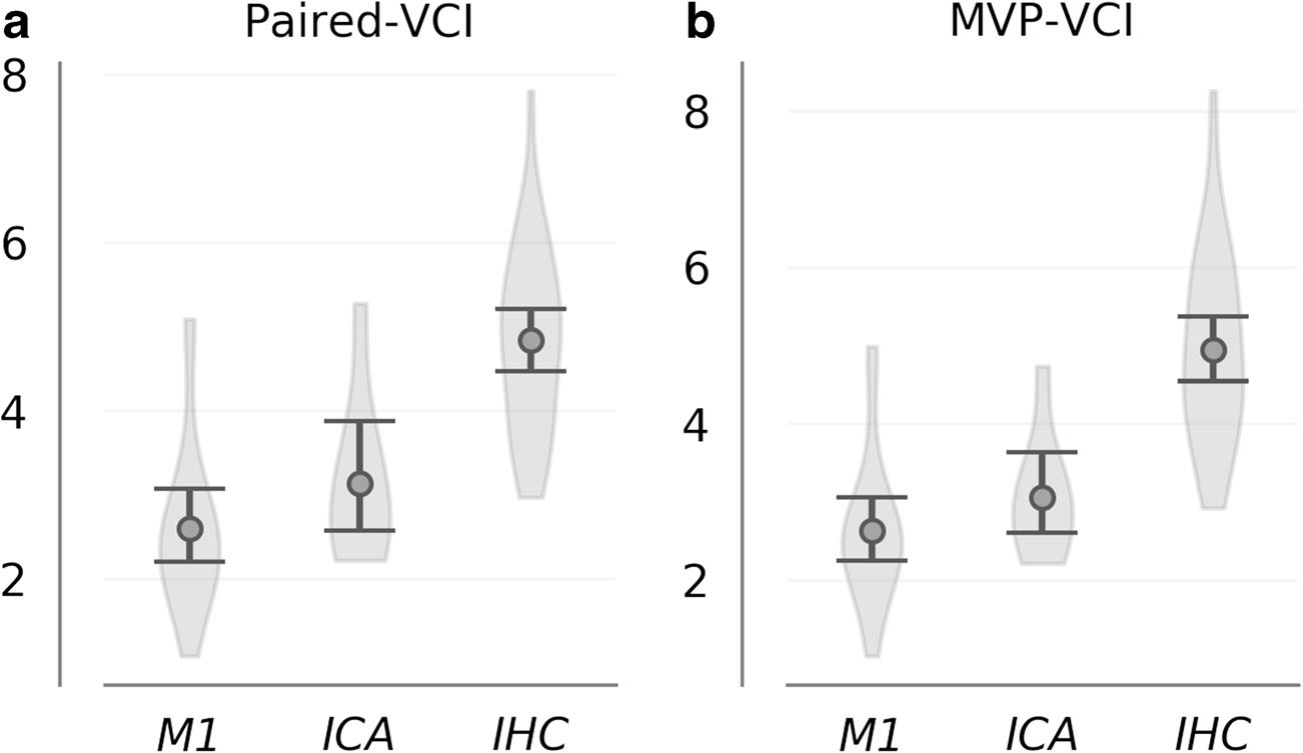
Paired-VCI (**a**) and MVP-VCI (**b**) curvature distributions are shown for subgroups corresponding to occlusion location; M1 (*N* = 20) vs ICA (*N* = 8) occlusions. While observed differences between M1 and ICA subgroups did not reach significance, those observed between each subgroup and corresponding IHC controls were highly significant; both for paired-VCI as well as MVP-VCI

When paired-VCI distributions were broken into subgroups according to occlusion location, group means were 2.6 (CI = 2.9–3.1), and 3.14 (CI = 2.6–3.9) for the M1 and ICA subgroups, respectively (Fig. 4a). Similarly, group means for MVP-VCI subgroups were 2.6 (CI = 2.3–3.1) for M1 and 3.06 (CI = 2.6–3.6) for ICA (Fig. 4b). While a trend was evident for greater curvature in the ICA subgroup relative to M1 (*p* = 0.07 for paired-VCI; *p* = 0.09 for MVP-VCI), the observed effects did not reach significance. Differences between occlusion subgroups were indeed very small relative to differences between each subgroup and corresponding controls, which were significant for all four comparisons (*p* ≪ 0.001 for M1 vs. IHC, and ICA vs IHC; for both paired-VCI and MVP-VCI).

## Discussion

Our results demonstrate that VCI is a robust metric for detecting LVO, with a number of important advantages relative to current heuristic procedures. Such methods require acquisition of CBFV and PMD waveforms from multiple vessels in each hemisphere, which must be obtained and evaluated by highly trained personnel with advanced anatomical knowledge. VCI has powerful predictive utility when measured from a single recording of MCA flow, which is significantly enhanced by a paired bilateral recording, regardless of inter-hemispheric depth disparity or occlusion location. It is computable in real time and is easily understood and communicated. Most importantly, VCI biomarker performance considerably exceeds current prehospital stroke assessment scales [9], which a recent review of clinical LVO prediction instruments concludes are unlikely to predict LVO with both high sensitivity and specificity [10]. The evidence presented here suggests VCI could provide the platform for development of a highly accurate, automated, prehospital LVO detection system.

Previous studies assessing the validity of TCD in detecting LVO have demonstrated consistent results with which our current findings align. Sloan et al. (2004) aggregated results over several studies comparing TCD to various angiographic methodologies, reporting sensitivity and specificity ranging from 85 to 95%, and 90 to 98% for MCA occlusions, but generally lower values for occlusions of other arteries, including ICA [28]. Specifically, Demchuk et al. (2000) demonstrated sensitivity and specificity of their TCD assessment procedure of 83% and 94%, though sensitivity for the MCA and proximal segment of the ICA were higher (93% and 94%, respectively) [29]. More recent studies comparing TCD to CTA corroborate this range. Tsivgoulis and colleagues [16] used a procedure to detect occlusions or stenoses based primarily on the presence of the pathological waveform morphologies described by Demchuk et al. [13], reporting sensitivity and specificity of 79% and 94%. Brunser et al. [17] used a similar procedure, but with additional incorporated power M-mode criteria, reporting sensitivity and specificity of 90% and 94% for the presence of occlusion in any artery. The sensitivity/specificity ranges observed for our paired-VCI biomarker compare well with these results, especially when considering maximal velocity pairs. It is important to note, however, that heuristic methods perform well because information is meticulously extracted from bilateral comparisons of flow direction, velocity, and morphology across multiple vessels (MCA, PCA, ACA, ICA). Here, we have shown that VCI contains diagnostic information on par with such assessments given only bilateral measurements of the MCA, which is bolstered by probing across multiple depths. This is advantageous because the MCA is the most easily insonated intracranial artery, with the longest expected segment of measurable depths.

### Pair Selection and Free Parameters

In this work, we have estimated the uncertainty associated with computing the VCI biomarker from random bilateral measurements of the MCA (paired-VCI) and demonstrated that a simple pair selection scheme serves to further increase biomarker efficacy (MVP-VCI). This result obviates the question as to what degree the pair selection criterion might be further optimized. The question is especially relevant for distal MCA occlusions for which cerebral hemodynamics might vary considerably across depths in the vicinity of the occlusion. Indeed, since the M2 segment typically ranges from 30 to 40 mm, it is possible that extending the sampled depth range could allow us to capture additional relevant dynamics. Another candidate pair selection method that will be investigated in future work involves looking across depths for velocity “discontinuities” which might be indicative of disrupted flow. In such cases, the highest velocity waveform would not be the most information rich concerning the presence of pathology. However, the success of such schemes depends critically on the availability of data across multiple depths, which can be constrained both by subject anatomy and available scan time.

Another potential optimization of the biomarker computation involves the choice of the free parameter defining the beat canopy (see “TCD Waveform Processing” section). The purpose of the parameter is to exclude noise prevalent near the waveform diastole. A thorough analysis of VCI dependence on this parameter is outside the scope of this work. However, in practice, we find that as long as the parameter is not set too high (so as to exclude important morphological structure), the results do not change appreciably. For example, when comparing our current results to those obtained by re-running the full suite of analyses with the free parameter set to zero (thereby specifying the beat “canopy” to include *all* points of the waveform), the 94.2% AUC observed for VCI from max velocity pairs falls only to 93.7%, the associated 90.9% sensitivity falling to 87.9%, with identical specificity (87.9%). Future work will investigate whether refinement of the canopy definition might further improve performance.

### Curvature and Morphology

**Fig. 5:**
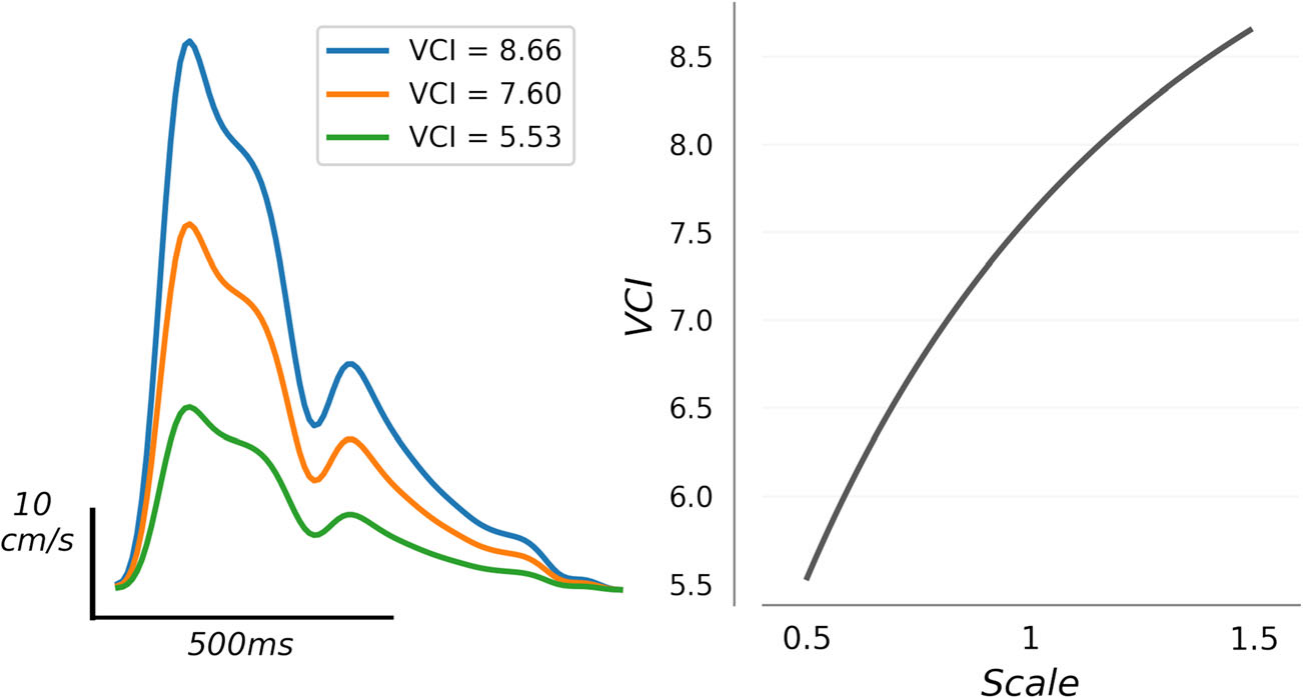
An example waveform (orange line, left column) is shown along with scaled versions of the same waveform indicated in green (scale factor 0.5), and blue (scale factor 1.5). The relation is depicted graphically in the right column, where VCI is computed for intermediate scales. The relation determines that when occlusion acts to dampen the waveform, this effect will be reflected in the VCI biomarker

VCI is effective as a diagnostic metric because it captures much of the same information implicit in the morphological labels currently in use, i.e., minimal, blunted, and dampened flows. Indeed, curvature is greatest when CBFV waveforms show multiple, pronounced, complementary peaks and valleys (Fig. 1). For the *minimal* waveform described by Demchuk et al. [13], our smoothing/averaging procedure effectively guarantees near-zero curvature. Their *blunted* waveform, characterized by delayed flow acceleration with maximum velocity in mid-to-late systole, lacks a well-defined early systolic peak and associated valley, and thus possesses an inherently smoother systolic complex with lower relative curvature. *Dampened* waveforms, defined by normal morphology but lower velocity relative to the unaffected side, also possess lower curvature relative to a paired higher velocity waveform of identical shape (Fig. 5). It is important to note that, however useful, these pathological categories cannot fully characterize the complete spectrum of variance in waveform morphology. Two waveforms can be comparatively more or less blunted, for example, and a single waveform can be both blunted and dampened. VCI provides a straightforward way of both unifying and quantifying these categories.

One potential complication which could conceivably impact the biomarker computation relates to abnormal heart rhythms such as those arising from atrial fibrillation and ectopic heartbeat. However, such cases are not particularly difficult to deal with, especially when pathological rhythms are inconsistent. Irregular timing between beats is not problematic, as simple outlier detection can readily identify beats of anomalous length/shape and exclude them from the ensemble average. The situation is slightly more difficult when such phenomenon occurs with high regularity throughout the recording interval. If inter-beat intervals are irregular enough, then temporal normalization may be required to optimize reconstruction of the average morphology. Though no such cases were encountered in this initial data, precise decision criterion dictating when such steps are required, and how they might be optimally implemented, must be addressed as further data is acquired.

Some limitations of our study and directions for future work should be noted. First, we acknowledge the possibility that the higher subject loss post-enrollment in the LVO group could potentially introduce bias in our sample. Further data is clearly required to better estimate VCI variability and to provide power and granularity for myriad important subgroup analyses (age, gender, occlusion type, etc.). Additionally, much work is needed to clarify the physiological underpinnings of occlusion-related changes in waveform morphology. A more complete theoretical understanding would help optimize diagnoses, especially when insonating the immediate neighborhood of an occlusion, where hemodynamics may show a broader array of flow behaviors. While occlusion certainly disturbs flow mechanically, by effectively decreasing local vessel cross-sectional area, our results clearly demonstrate impacted curvature measurable in the cerebral hemisphere *contralateral* to occlusion. The manner in which such local disturbances propagate throughout the brain is currently unknown, but could be clarified in future work via empirical/mathematical simulation using realistic models of human vasculature. Armed with better understanding, VCI might readily be adapted for assessment of other cerebrovascular pathologies, such as atherosclerosis, elevated intracranial pressure, and traumatic brain injury.

### Conclusions

VCI is an analytically valid metric for assessing the presence of LVO, with results comparable to those obtained by expert evaluation of laborious multi-vessel protocols. As a metric, it provides a means of quantifying the morphological categories currently used for classification of stroke pathology. For clinical purposes, VCI sensitivity can be adjusted by informed specification of the diagnostic threshold and easily calibrated as future data is obtained. It can be assessed from a single MCA recording, or supplemented by paired bilateral recordings for increased statistical power, regardless of occlusion location or inter-hemispheric depth disparity. It is easily computed and readily understandable, making it an ideal candidate biomarker for field-deployable diagnostic TCD systems in the future.

## References

[1] Benjamin EJ, Blaha MJ, Chiuve SE (2017). Heart disease and stroke statistics-2017 update: a report from the American Heart Association. Circulation.

[2] National Institute of Neurological Disorders and Stroke rt-PA Stroke Study Group (1995). Tissue plasminogen activator for acute ischemic stroke. N Engl J Med..

[3] Saqqur M, Uchino K, Demchuk AM, Molina CA, Garami Z, Calleja S, Akhtar N, Orouk FO, Salam A, Shuaib A, Alexandrov AV, for CLOTBUST Investigators (2007). Site of arterial occlusion identified by transcranial Doppler predicts the response to intravenous thrombolysis for stroke. Stroke.

[4] Saver JL, Goyal M, van der Lugt A, Menon BK, Majoie CBLM, Dippel DW, Campbell BC, Nogueira RG, Demchuk AM, Tomasello A, Cardona P, Devlin TG, Frei DF, du Mesnil de Rochemont R, Berkhemer OA, Jovin TG, Siddiqui AH, van Zwam WH, Davis SM, Castaño C, Sapkota BL, Fransen PS, Molina C, van Oostenbrugge RJ, Chamorro Á, Lingsma H, Silver FL, Donnan GA, Shuaib A, Brown S, Stouch B, Mitchell PJ, Davalos A, Roos YBWEM, Hill MD, for the HERMES Collaborators (2016). Time to treatment with endovascular thrombectomy and outcomes from ischemic stroke: a meta-analysis. JAMA.

[5] Sheth SA, Jahan R, Gralla J, Pereira VM, Nogueira RG, Levy EI, Zaidat OO, Saver JL, for the SWIFT-STAR Trialists (2015). Time to endovascular reperfusion and degree of disability in acute stroke. Ann Neurol.

[6] Goyal M, Jadhav AP, Bonafe A, Diener H, Mendes Pereira V, Levy E, Baxter B, Jovin T, Jahan R, Menon BK, Saver JL, For the SWIFT PRIME investigators (2016). Analysis of workflow and time to treatment and the effects on outcome in endovascular treatment of acute ischemic stroke: results from the SWIFT PRIME randomized controlled trial. Radiology.

[7] Kelly KM, Holt KT, Neshewat GM, Skolarus LE (2017). Community interventions to increase stroke preparedness and acute stroke treatment rates. Curr Atheroscler Rep.

[8] Smith EE, Saver JL, Cox M, Liang L, Matsouaka R, Xian Y, Bhatt DL, Fonarow GC, Schwamm LH (2017). Increase in endovascular therapy in get with the guidelines-stroke after the publication of pivotal trials. Circulation.

[9] Hastrup S, Damgaard D, Andersen G (2016). Prehospital acute stroke severity scale to predict large artery occlusion: design and comparison with other scales. Stroke.

[10] Smith EE, Kent DM, Bulsara KR, et al. Accuracy of prediction instruments for diagnosing large vessel occlusion in individuals with suspected stroke: a systematic review for the 2018 guidelines for the early management of patients with acute ischemic stroke. Stroke 2018:STR.0000000000000160. 10.1161/STR.0000000000000160 10.1161/STR.0000000000000160 29367333

[11] Alexandrov AV, Sloan MA, Tegeler CH, Newell DN, Lumsden A, Garami Z, et al. Views and reviews practice standards for transcranial Doppler ( TCD ) ultrasound . Part II. Clinical indications and expected outcomes. 2010;22:215–24. 10.1111/j.1552-6569.2010.00523.x. 10.1111/j.1552-6569.2010.00523.x 20977531

[12] Alberts MJ, Latchaw RE, Selman WR, Shephard T, Hadley MN, Brass LM, Koroshetz W, Marler JR, Booss J, Zorowitz RD, Croft JB, Magnis E, Mulligan D, Jagoda A, O'Connor R, Cawley CM, Connors JJ, Rose-DeRenzy JA, Emr M, Warren M, Walker MD, for the Brain Attack Coalition (2005). Recommendations for comprehensive stroke centers: a consensus statement from the brain attack coalition. Stroke.

[13] Demchuk AM, Christou I, Wein TH, Felberg RA, Malkoff M, Grotta JC, Alexandrov AV (2000). Specific transcranial Doppler flow findings related to the presence and site of arterial occlusion. Stroke.

[14] Alexandrov AV, Demchuk AM, Burgin WS (2002). Insonation method and diagnostic flow signatures for transcranial power motion (M-mode) Doppler. J Neuroimaging.

[15] Saqqur M, Hill MD, Alexandrov AV, Roy J, Schebel M, Krol A, Garami Z, Shuaib A, Demchuk AM (2006). Derivation of power M-mode transcranial doppler criteria for angiographic proven MCA occlusion. J Neuroimaging.

[16] Tsivgoulis G, Sharma VK, Lao AY, Malkoff MD, Alexandrov AV (2007). Validation of transcranial Doppler with computed tomography angiography in acute cerebral ischemia. Stroke.

[17] Brunser AM, Lavados PM, Hoppe A, Lopez J, Valenzuela M, Rivas R (2009). Accuracy of transcranial doppler compared with CT angiography in diagnosing arterial obstructions in acute ischemic strokes. Stroke.

[18] Moehring MA, Spencer MP (2002). Power M-mode Doppler (PMD) for observing cerebral blood flow and tracking emboli. Ultrasound Med Biol.

[19] Kurji A, Debert CT, Whitelaw WA, Rawling JM, Frayne R, Poulin MJ (2006). Differences between middle cerebral artery blood velocity waveforms of young and postmenopausal women. Menopause.

[20] Aggarwal S, Brooks DM, Kang Y, Linden PK, Patzer JF (2008). Noninvasive monitoring of cerebral perfusion pressure in patients with acute liver failure using transcranial doppler ultrasonography. Liver Transplant.

[21] Lockhart CJ, Gamble AJ, Rea D, Hughes S, McGivern RC, Wolsley C, Stevenson M, Harbinson MT, Plumb RD, McVeigh GE (2006). Nitric oxide modulation of ophthalmic artery blood flow velocity waveform morphology in healthy volunteers. Clin Sci (Lond).

[22] Crutchfield KE, Razumovsky AY, Tegeler CH, Mozayeni BR (2004). Differentiating vascular pathophysiological states by objective analysis of flow dynamics. J Neuroimaging.

[23] Kim S, Hamilton R, Pineles S, Bergsneider M, Hu X (2013). Noninvasive intracranial hypertension detection utilizing semisupervised learning. IEEE Trans Biomed Eng.

[24] Group F-NBW. BEST (Biomarkers, EndpointS, and Other Tools) Resource. Food and Drug Administration (US); 2016. http://www.ncbi.nlm.nih.gov/pubmed/27010052. Accessed 5 Feb 2018. 27010052

[25] Efron B, Tibshirani RJ (1993). An Introduction to the Bootstrap. Monograps on statistics and applied probability. 57th ed.

[26] Newell DW, Aaslid R, Newell DW, Aaslid R (1992). Transcranial Doppler.

[27] Youden W (1950). Index for rating diagnostic tests. Cancer.

[28] Sloan MA, Alexandrov AV, Tegeler CH, Spencer MP, Caplan LR, Feldmann E, Wechsler LR, Newell DW, Gomez CR, Babikian VL, Lefkowitz D, Goldman RS, Armon C, Hsu CY, Goodin DS, Therapeutics and Technology Assessment Subcommittee of the American Academy of Neurology (2004). Assessment: transcranial Doppler ultrasonography: report of the Therapeutics and Technology Assessment Subcommittee of the American Academy of Neurology. Neurology.

[29] Demchuk AM, Christou I, Wein TH, Felberg RA, Malkoff M, Grotta JC, Alexandrov AV (2000). Accuracy and criteria for localizing arterial occlusion with transcranial Doppler. J Neuroimaging.

